# Decrease of *FZD4* exon 1 methylation in probands from *FZD4*-associated FEVR family of phenotypic heterogeneity

**DOI:** 10.3389/fmed.2022.976520

**Published:** 2022-10-24

**Authors:** Miaomiao Liu, Jia Luo, Huazhang Feng, Jing Li, Xiang Zhang, Peiquan Zhao, Ping Fei

**Affiliations:** Department of Ophthalmology, Xinhua Hospital Affiliated to Shanghai Jiao Tong University School of Medicine, Shanghai, China

**Keywords:** familial exudative vitreoretinopathy (FEVR), the receptor frizzled-4 (FZD4), phenotypic heterogeneity, DNA methylation, epigenetics

## Abstract

Familial exudative vitreoretinopathy (FEVR) is an important cause of childhood blindness and is clinically characterized by phenotypic heterogeneity. FEVR patients harboring the same genetic mutation vary widely in disease severity. The purpose of this study was to explore non-genetic factors that regulate FEVR phenotypic heterogeneity. We detected methylation levels of 21 CpG sites located at the *FZD4* exon 1 region of 11 probands, 12 asymptomatic/paucisymptomatic carriers and 11 non-carriers from 10 unrelated *FZD4*-associated FEVR families using bisulfite amplicon sequencing (BSAS). Our results showed reduced methylation level of *FZD4* exon 1 in probands, suggesting that *FZD4* exon 1 methylation level may be negatively linked with FEVR disease severity. It provided a new research direction for follow-up research, helping us better understand the complexity of the FEVR-causing mechanism.

## Introduction

Familial exudative vitreoretinopathy (FEVR) is a clinically and genetically heterogeneous ophthalmic disorder characterized by incomplete retinal vascular development. The disease was first described in 1969 by Criswick and Schepens (1), and it is a major cause of vision loss in juveniles. Several sight-threatening complications are considered secondary to retinal avascularity, including retinal neovascularization and exudates, retinal fold and detachments, vitreous hemorrhage, and macular ectopia, ultimately leading to total blindness. The clinical appearance of FEVR varies considerably. The mildly affected patients may have only a small avascular area at the peripheral retina, which is visible only by fundus fluorescein angiography (FFA), while severely affected patients often registered as blind during infancy. With extensive fundus screening, we now realize that the prevalence of FEVR is much higher than we thought before (2). Therefore, it is imperative to study the mechanism underlying FEVR in order to seek for advanced therapy.

In the past decades, research on FEVR has mainly focused on genomics. At least 9 genes have been reported to cause FEVR: norrin cystine knot growth factor *NDP* (*NDP*; MIM: 300658) (3); low-density lipoprotein receptor-related protein 5 (*LRP5*; MIM: 603506) (4), tetraspanin 12 (*TSPAN12*; MIM: 613138) (5), frizzled class receptor 4 (*FZD4*; MIM: 604579) (6, 7), kinesin family member 11 (*KIF11*; MIM:148760) (8, 9), zinc finger protein 408 (*ZNF408*; MIM: 616454) (10), catenin, β-1 (*CTNNB1*; MIM: 116806) (11, 12), jagged 1 (*JAG1*; MIN: 601920) (13), and catenin, α-1 (*CTNNA1*; MIM:116805) (14). These gene mutations explain approximately 50% of FEVR cases. Mutations in six of the nine genes (*FZD4, TSPAN12, NDP, LRP5, CTNNB1*, and *CTNNA1*) are related to the Norrin/ Wnt β-catenin signaling pathway, which coordinates retinal development and angiogenesis by regulating cell survival, differentiation, proliferation, and migration (15, 16). The *NDP* gene encodes the extracellular ligand Norrin, which binds specifically to the receptor/coreceptor (*FZD4, LRP5* and *TSPAN12*) and forms a ligand-receptor complex. Upon activation of Norrin signaling, β-catenin translocates to the nucleus, binds to *TCF/LEF* (T cell factor/lymphoid-enhancing factor) and subsequently modulates gene expression (17–19). Mutations in *CTNNA1* induce overactivation of Norrin/β-catenin signaling and interrupt cell junctions (20, 21). The inheritance patterns of FEVR include autosomal dominant (AD), autosomal recessive (AR), and X-linked recessive (XL), with AD being the most common mode. Despite clear definition of causative genes, mutations of the same gene often manifest with drastic phenotypic heterogeneity in carriers (2, 22, 23). First-degree relatives of index patients with severe FEVR are often asymptomatic despite carrying the same mutation (24). This suggests that other risk factors beyond genetic mutation may contribute to FEVR pathogenesis.

Gene expression variability is one of the important causes for phenotypic variation. Epigenetic modification affects gene expression. One of the best understood epigenetic modification is DNA methylation. DNA methylation usually occurs in the cytosine guanine (CpG) dinucleotide. It affects gene expression by changing chromatin structure, DNA conformation, DNA stability and protein accessibility to DNA (25, 26). CpG islands are regions in the genome where CpG levels are significantly higher than the average CpG levels of the whole gene. CpG islands usually are found in the promoter or exon 1 region. Although few studies addressed DNA methylation change in FEVR, methylation changes have been demonstrated in other ocular vitreoretinopathy diseases such as age-related macular degeneration (AMD), proliferative vitreoretinopathy (PVR), and diabetic retinopathy (DR) (27–30). Therefore, we hypothesized that DNA methylation changes exist in FEVR causative genes, and such changes could affect gene expression and leading to FEVR phenotypic heterogeneity.

In this study, 10 separate *FZD4*-associated FEVR families were recruited to investigate the *FZD4* methylation status of FEVR probands and their asymptomatic/paucisymptomatic family members carrying the same mutation. Our results showed reduced *FZD4* exon 1 methylation in probands, suggesting that *FZD4* exon 1 methylation level may be negatively linked with FEVR disease severity. To the best of our knowledge, this is the first paper that attempts to investigate the association between DNA methylation and heterogeneous presentation of FEVR.

## Methods

### Subjects

760 probands were recruited based on a clinical diagnosis of FEVR at the ophthalmology department of Xinhua hospital from May 2010 to August 2019. Next Generation Sequencing (NGS) revealed that 112 probands carried only *FZD4* gene mutations, without other known FEVR-associated gene mutation. We further collected family history information of these 112 *FZD4* Gene mutation probands. Finally, 10 *FZD4*-associated FEVR families met the following inclusion criteria were recruited. each enrolled family contained at least one FEVR proband (Staged 3–5), one asymptomatic/ paucisymptomatic carrier of the mutant allele (clinically healthy or staged 1) and one non-carrier. Stage 2 individuals were excluded to maximize the phenotypic heterogeneity feature in our study design. Clinical staging was based on Trese's 5-stage system (31) and the severity of patients was determined by the highest stage of FEVR in either eye. Subjects with histories of oxygen inhalation and preterm birth were also excluded. all enrolled participants were diagnosed by ophthalmologist with expertise in FEVR Based on clinical manifestations, genotyping, fundus examination and FFA. This study was approved by the Institutional Review Board of Xin Hua Hospital Affiliated to Shanghai Jiao Tong University School of Medicine. All work was performed in accordance with the approved study protocol. written informed content was obtained from all the participants or their parents.

### Analysis of *FZD4* Rs10128621 single nucleotide polymorphisms

Cis-regulatory variants might also modify mutation penetrance and contribute to clinical heterogeneity (32). *FZD4* rs10128621 SNP is the only known SNP which could affect FZD4 gene expression (33). We performed Sanger sequencing to analyze *FZD4* rs10128621 SNP in our cohort. The primer sequences were as follows: 5'-ACCTTGGCTTCCTAAAGTGTTG-3' (forward primer) and 5'-CTGCCTGAGAGACAGAGTAAGA-3' (reverse primer). The expected amplicon is 297 bp in length.

### Bisulfite amplicon sequencing for methylation analysis

DNA methylation was analyzed by Bisulfite amplicon sequencing (BSAS). About 5 mL of peripheral blood samples were collected from each participant in a standard EDTA tube. Genomic DNA was extracted and purified using QIAamp^®^ DNA Mini kit. One hundred thirty microliter of CT Conversion Reagent was added to 300 ng purified DNA samples as per the manufacturer's instructions. This process converted unmethylated cytosines (C) in CpG sites to uracil (U), whereas methylated C remained C. The primer sequences were as follows: 5′-TTTAGTTTGGGGTTGTTTTTGTAGT-3′ (forward primer) and 5′-CTTTTCCAAAAAAACTATCTCCTTC-3′ (reverse primer). PCR library was constructed using VAHTS Turbo DNA Library Prep Kit for Illumina and sequenced on a Miseq system (Illumina). Quality control was performed using FastQC v 0.11.2. Mapping and calling of the methylation levels were performed using bismark software and methylKit (R package 4.1.0), respectively.

### Statistical analysis

Independent Samples *T*-test was used to pairwise compare the methylation differences among probands, carriers and non-carriers. Significant differences were determined as *p* < 0.05. Data analysis was performed using Graphpad 9.0.

## Results

### Characteristics of subjects

**Figure 1:**
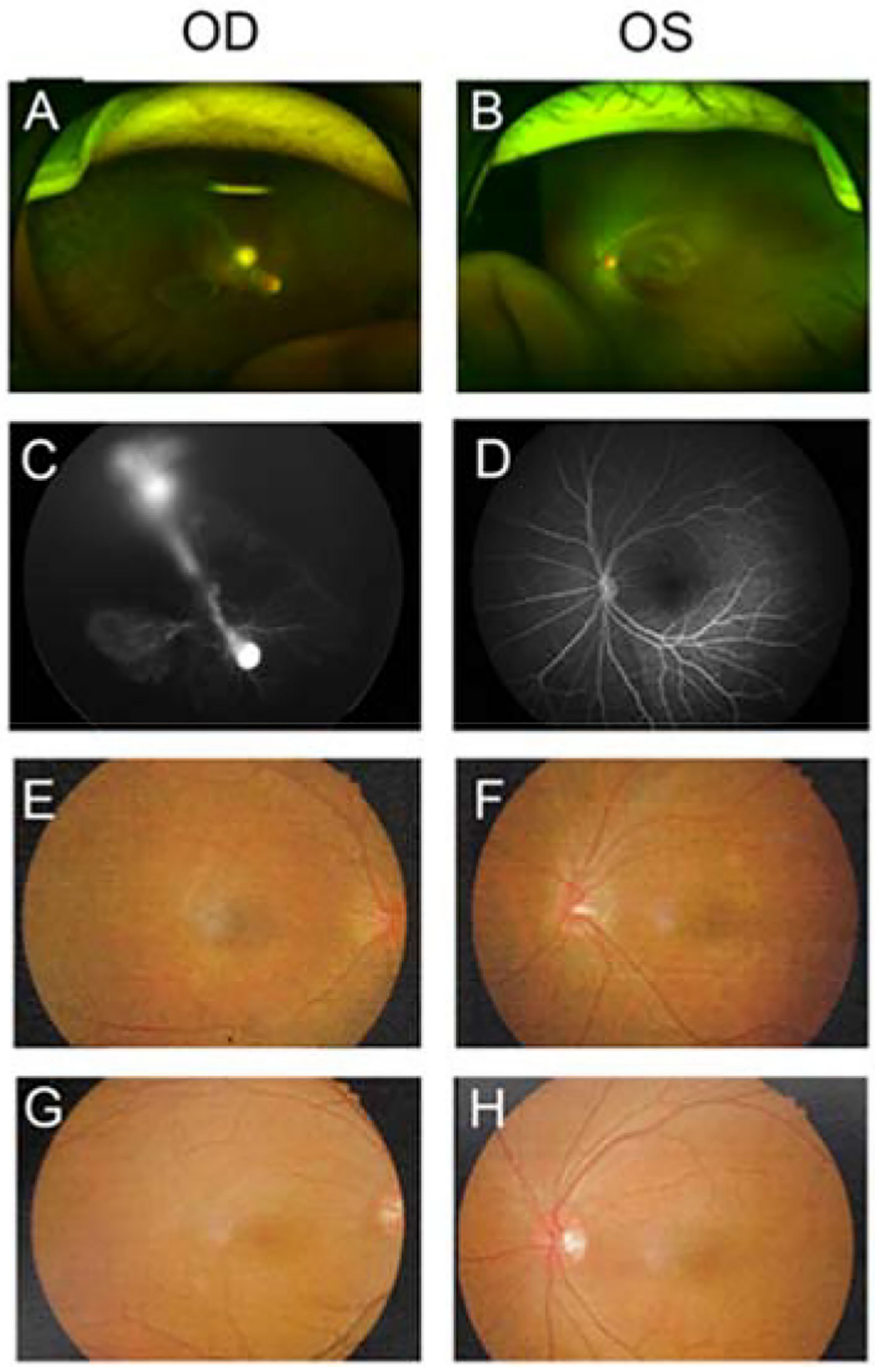
Fundus photographs and fundus fluorescein angiography (FFA) of subjects from family No. 8. In this family, the c.205C>T mutation was detected in a 4-year-old proband boy and it was inherited from his father. OD and OS represented right eye and left eye, respectively. **(A–D)** Fundus and FFA photographs of FEVR proband. The proband suffered from monocular FEVR disease in the right eye and was staged 4A. Severe fibrous proliferation membrane connected the optic disc to the lens in the right eye, with subtotal retinal detachment and peripheral avascular zone. The left eye exhibited normal retinal appearance without prolonged filling time or leakage. **(E,F)** Fundus photographs of father. Fundus examination didn't reveal any FEVR presentation although the father carried the c.205C>T mutation. **(G,H)** Fundus photographs of mother. The mother was a non-carrier without any fundus abnormality.

Thirty-four members of 10 *FZD4*-associated FEVR families were recruited in our study, including 11 FEVR probands, 12 asymptomatic/paucisymptomatic carriers of the same mutation, and 11 non-carriers of the mutant alleles. FEVR probands and family members were studied as a whole to best ensure similar genetic and environmental backgrounds. While FEVR probands had the most advanced ocular manifestations early in life, most of the family members carrying the same gene mutation were asymptomatic, only 4 carriers presented with Stage 1 FEVR. The mean age of probands, carriers and non-carriers were 35.1 ± 48.5 months (average ± standard deviation), 32.2 ± 12.2 years and 32.5 ± 14.8 years, respectively. Other than mutations in *FZD4*, no additional known FEVR-associated mutation was found in these subjects. Family No. 2, 5, 8 (chr11-86665923 c.205C>T) and family No. 7, 10 (chr11: 86665894-86665911 c.217_234del) shared the same mutation, respectively. The clinical details and genetic test results were summarized in Table 1. Fundus photographs and FFA of family No. 8 were presented in Figure 1.

**Table 1:** Clinical details and genetic test results of 34 subjects from 10 FEVR families.

**Family ID**	**FEVR probands**						**Asymptomatic/paucisymptomatic**		**Non carrier**	**Genomic coordinate** **(hg19)**	**Exon**	**Nucleotide change**	**Zygosity**
	**Gender**	**Age at diagnose /month**	**Disease stage (OD/OS)**	**Retinal folds** **(OD/OS)**	**Retinal** **detachment** **(OD/OS)**	**Others**	**Relation**	**Disease stage**					
1	M	42	5/1	NA/No	NA/No	OD corneal staphyloma	Father	N	Mother	chr11-86662767	Exon2	c.1031dup	het
2	M	12	3b/5a	NA	NA		Mother	N	Father	chr11-86665923	Exon1	c.205C>T	het
3	F	12	1/5	No/No	No/Yes	OS disappearance of anterior chamber	Mother	N	Father brother	chr11-86663328	Exon2	c.470T>C	het
4	M	29	4a/4a	Yes/Yes	Yes/Yes		Mother	N	Father	chr11-86663485	Exon2	c.313A>G	het
5	F	19	5/1	NA	NA		Mother	N	Father	chr11-86665923	Exon1	c.205C>T	het
6	M	6	1/5	No/Yes	No/Yes	OS disappearance of anterior chamber OS vitreous hemorrhage	Father grandfather	1 1	Grandmother	chr11:86662513–86662516	Exon2	c.1282_1285del	het
	F	174	1/5	No/Yes	No/Yes								
7	M	17	5/0	Yes/No	Yes/No		Mother	1	Father	chr11:86665894–86665911	Exon1	c.217_234del	het
8	M	54	4a/0	Yes/No	Yes/No	OD lens opacity	Father	N	Mother	chr11-86665923	Exon1	c.205C>T	het
9	M	5	4a/4a	Yes/Yes	Yes/Yes		Father	1	Mother	chr11-86662817	Exon2	c.981G>A	het
10	M	16	4a/3a	Yes/Yes	Yes/Yes		Father brother	1 N	Mother	chr11:86665894–86665911	Exon1	c.217_234del	het

M, male; F, female; N, normal fundus; het, heterozygous; OD, right eye; OS, left eye; NA, not available.

### Map of CpG island in the *FZD4* exon 1 region

**Figure 2:**
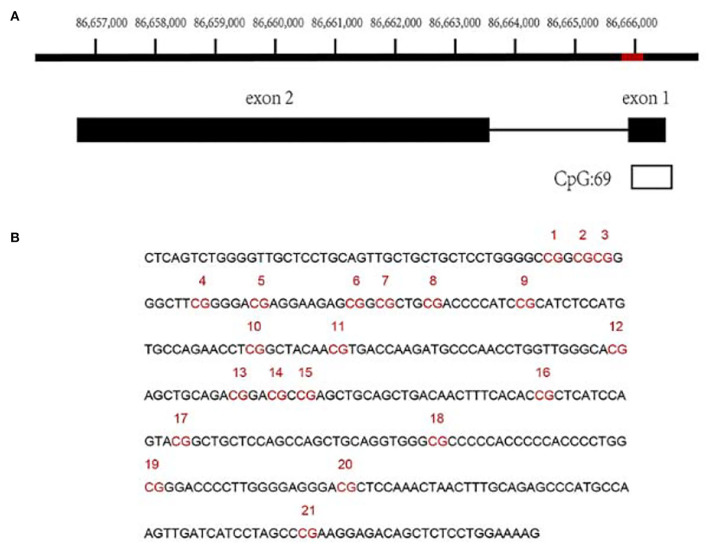
Analysis of methylation of CpG sites in the FZD4 exon 1 region. **(A)** The red region showed the location of tested sequence. The black box showed FZD4, black line indicated intergenic region and the white box indicated the CpG island region (CpG: 69) located in the exon 1 region of FZD4. **(B)** The sequence and CpG sites analyzed in this study were presented. The CpG sites were numbered and indicated as red.

We confirmed the methylation of CpG islands in *FZD4* using a genomic map of *FZD4* (NM_012193.4). *FZD4* is located on the negative strand of chromosome 11. Most of the CpG island (CpG:69) exists in the exon 1 region. Twenty-one CpG sites were identified from a fragment in the exon 1 region at 86,665,722–86,666,073 (Figure 2).

### Methylation analysis of 21 CpG sites

Before DNA methylation, we performed Sanger sequencing to analyze *FZD4* rs10128621 SNP in our cohort. Sanger sequencing results showed a complete consistency of G base. Our results did not support the role of *FZD4* rs10128621 SNP in the phenotypic heterogeneity of our FEVR patients. (Supplementary Figure S1)

**Figure 3:**
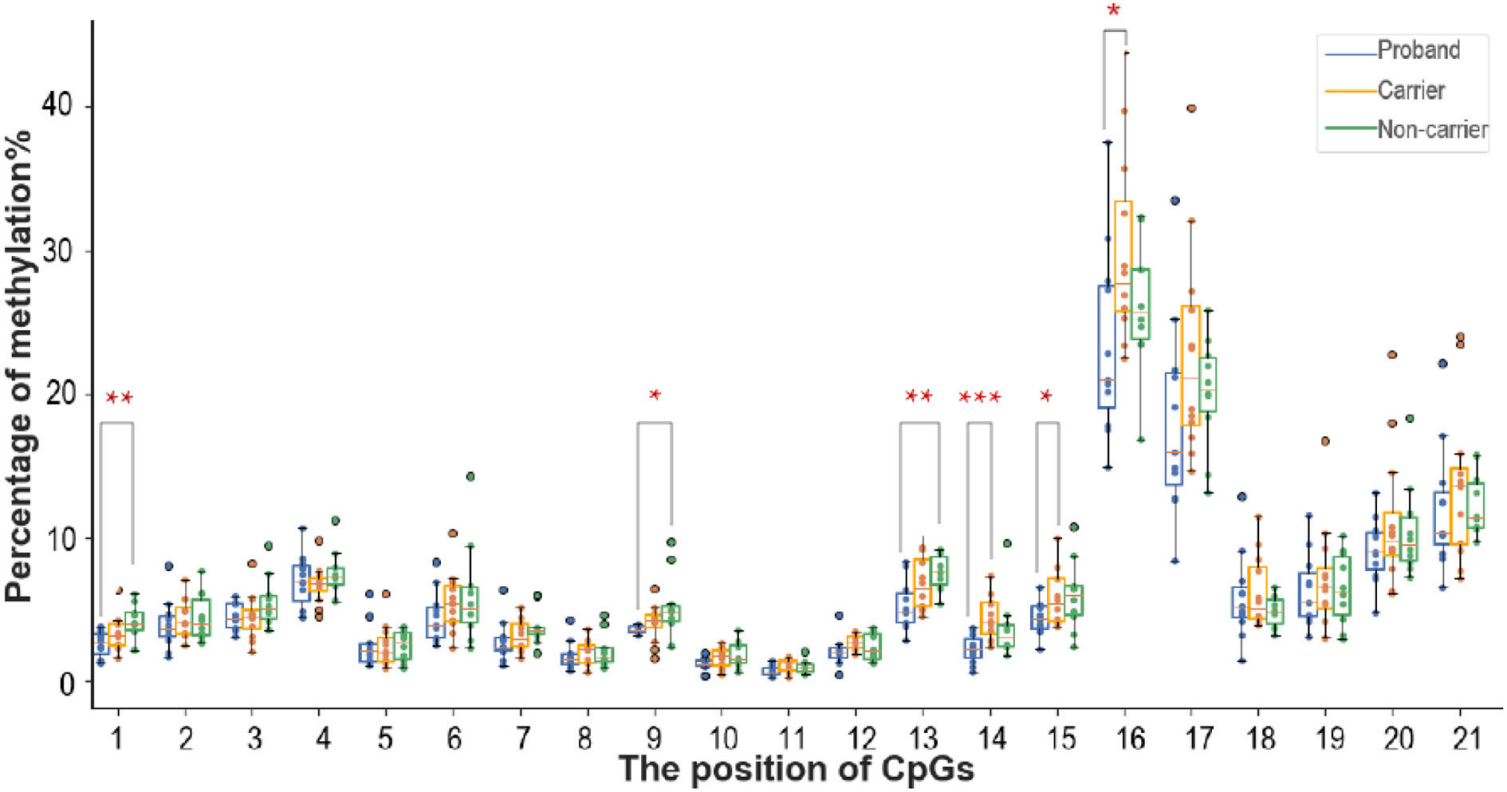
The graph showed methylation levels of 21 tested CpG sites. The horizontal axis represented the position of CpG sites and the vertical axis represented percentage of methylation. Blue, orange and green points respectively indicated to 11 FEVR probands, 12 carriers and 11 non-carriers. Box plot data represented range (error bars), median (horizontal line), quartile values (box) and outliers (dots). Median methylation levels of probands, carriers and non-carriers were compared in pair using student's *t*-test. DNA methylation levels of CpG1, CpG9 and CpG13 were significantly different between probands and non-carriers. DNA methylation levels of CpG14, CpG15 and CpG16 were significantly different between probands and carriers. *, *p* < 0.05; **, *p* < 0.01; ***, *p* < 0.001.

DNA methylation levels of 21 CpG sites identified above in probands and their family members were presented in Figure 3, except for the non-carrier of family No. 8, whose blood sample was not obtained due to data missing. At almost all tested CpG sites (19 out of 21), FEVR probands held the lowest average methylation level compared to carriers and non-carriers, except for CpG4 and CpG5, where probands had slightly higher methylation levels than carriers. At more than half of the CpG sites (12 out of 21), carriers held lower methylation levels than non-carriers. DNA methylation levels of probands were significantly lower than symptomatic/paucisymptomatic carriers in CpG14 (*p* = 0.0009), CpG15 (*p* = 0.0489) and CpG16 (*p* = 0.0320). DNA methylation levels of probands were significantly lower than non-carriers at CpG1 (*p* = 0.0009), CpG9 (*p* = 0.0371) and CpG13 (*p* = 0.0021). Overall, the results suggested probands had lower DNA methylation compared with carriers and non-carriers in *FZD4* exon 1 region.

**Figure 4:**
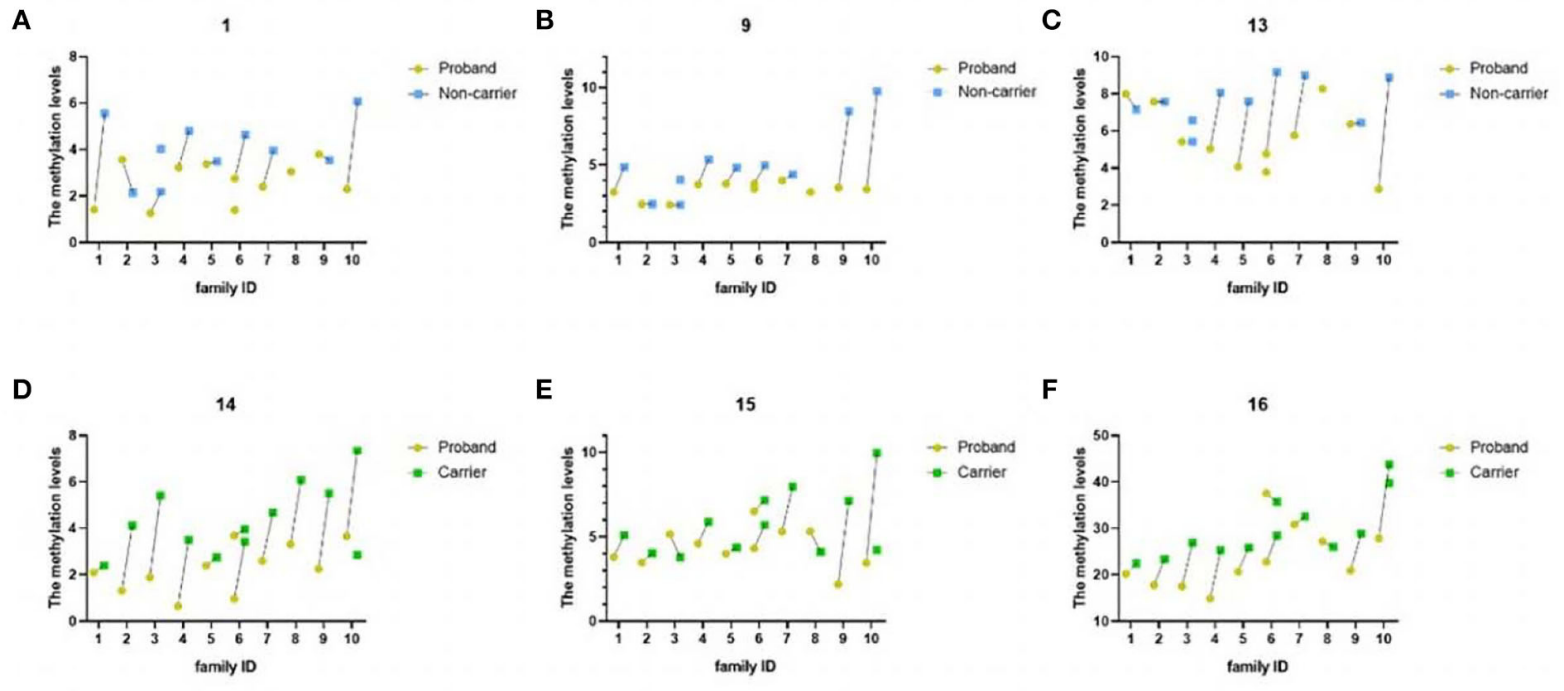
The figure above showed methylation levels at six significantly different CpG sites of 10 FEVR families. The horizontal axis represented family ID and the vertical axis represented the methylation level of specific CpG site. **(A–C)** DNA methylation levels of CpG1, CpG9 and CpG13 were significantly different between probands and non-carriers. Yellow and blue points indicated to probands and non-carriers, respectively. **(D–F)** DNA methylation levels of CpG14, CpG15 and CpG16 were significantly different between probands and carriers. Yellow and green points indicated to probands and carriers, respectively.

The methylation levels of these six significantly different CpG sites in each of the 10 FEVR families were shown in Figure 4. It showed that FEVR probands had lower methylation levels nearly in almost all cases. For example, at CpG14, probands in all 10 families had lower methylation levels. In addition, we noticed a negative correlation at CpG14 between methylation levels and disease severity within three families (No. 2, 5 and 8) that happened to carry the same mutation (chr11-86665923 c.205C>T). Proband from family No. 2 had the most advanced FEVR and the lowest methylation level at CpG14 compared to the rest. Similar negative correlations also existed for CpG15 and CpG16.

## Discussion

FEVR is a rare disease featured by peripheral retinal vascular abnormalities (19, 34). It is clinically characterized by phenotypic heterogeneity, suggesting that non-genetic factors are involved in disease etiology (2, 22, 23). Comparing the epigenetic features among FEVR family members with or without mutation would provide important information regarding epigenetic factors which could contribute to phenotypic heterogeneity. DNA methylation is a widely studied epigenetic modification type. Increasing number of evidence supported the role of DNA methylation in modulating the phenotypic expression of diseases (35–37). For example, Coppede et al. found that the increase of global DNA methylation might contribute to disease manifestation in amyotrophic lateral sclerosis (ALS) carriers of not fully penetrant SOD1 mutations (38). DNA methylation at promoter CpG islands (CGI) of Wnt pathway genes showed a significant association with progression-free survival in ovarian tumor patients (39). Although little is known about FEVR disease, altered DNA methylation has been demonstrated in many other vitreoretinopathy diseases such as age-related macular degeneration (AMD), proliferative vitreoretinopathy (PVR), and diabetic retinopathy (DR). Therefore, we hypothesized that DNA methylation changes might play a role in FEVR phenotypic heterogeneity.

Up to date, at least 9 genes have been reported to cause FEVR. In previous studies, we and others showed that with *FZD4* mutations accounting for the greatest proportion of FEVR in Chinese population (23, 40, 41). The *FZD4* gene encodes a 537–amino acid protein, which functions as a receptor of the Wnt signaling pathway (42). According to our previous work, more than half (51.43%) in the *FZD4* group were identified asymmetry, suggesting *FZD4* mutations might initiate the most diverse and asymmetric phenotypes (22). Previous study suggested epigenetic modifications might involve in the developing fetal retina which caused high asymmetric *FZD4* gene expression (43). Therefore, it is important to study non-genetic factors underlying *FZD4*-associated FEVR phenotypic heterogeneity.

Bisulfite sequencing was first reported by Frommer et al. in (44). The bisulfite treated-DNA is amplified by PCR, and then the cloning and sequencing results of PCR products are used to determine whether the CpG site was methylated. This method has been recognized as one of the most powerful approaches to investigate DNA methylation patterns due to its reliable results, high accuracy and high sensitivity. It allows the determination of methylation at a single-cytosine resolution and has become the “gold standard” for DNA methylation detection (45, 46).

The current study aimed to access evidence of a link between FEVR phenotypic heterogeneity and *FZD4* methylation status. We focused on 10 unrelated clinically heterogeneous FEVR families carrying only *FZD4* gene mutation. In this study, our goal was to investigate whether there was a difference in methylation levels between probands at severe FEVR stage and parents with mild phenotype. Stage 1 FEVR has only peripheral avascular zone seen by fluorescein fundus angiography. Patients with stage 1 FEVR may be stable for decades without any progression. Therefore, we grouped asymptomatic and stage 1 carriers together. Our data indicated a negative correlation between DNA methylation in *FZD4* exon 1 region and FEVR severity, which requires further validation. Among the six significantly different CpG sites, four sites (CpG13, CpG14, CpG15 and CpG16) were adjacent, suggesting that this region might play an important and unique role in the regulation of *FZD4* expression. Although the CpG9 of Family No. 9 and No.10 are obviously different, the probands still had the lowest average methylation level of the three groups and non-carriers had the highest average methylation level when these two families were eliminated. Our study suggests that changes in DNA methylation might contribute to the severity in *FZD4*-associated FEVR patients. The function of intragenic DNA methylation hasn't been well recognized. Brenet et al. found that DNA methylation at the first exon is tightly linked to transcriptional silencing (47), while others believed that the methylation in the body of genes can actually activate gene transcription (48, 49). Although it remains controversial, given that altered methylation of a specific CpG site might be enough to change gene expression [50], FEVR probands with reduced DNA methylation are highly likely to undergo altered gene expression, thus causing different degrees of disease severity.

This study is limited by the number of FEVR families enrolled. For 5 mutations analyzed here, only one family was found for each mutation. Alternatively, we took the approach of comparing the average overall methylation level in probands, carriers and non-carriers, which could have masked the effect of specific mutation on DNA methylation. In addition, age may be an independent factor in the methylation level. However, in this study, after excluding the “grandmother” and “grandfather” subjects in family 6, the probands still had the lowest average methylation level among the three groups, which is in line with our conclusion. What's more, we do not have RNA samples from our patients to validate the expression of *FZD4*. Future study with larger sample size is needed to validate our findings. Nevertheless, our results provided a new mechanism potentially responsible for phenotypic heterogeneity amongst FEVR patients.

## Data availability statement

The raw data supporting the conclusions of this article will be made available by the authors, without undue reservation.

## Ethics statement

The studies involving human participants were reviewed and approved by the Institutional Review Board of Xin Hua Hospital Affiliated to Shanghai Jiao Tong University School of Medicine. Written informed consent to participate in this study was provided by the participants' legal guardian/next of kin.

## Author contributions

ML and JLu are responsible for conceptualization, methodology, software, investigation, and writing original draft. HF is responsible for clinical data collection and writing. JLi and XZ are responsible for software and writing review. PF and PZ are responsible for supervision, writing review and funding acquisition. All authors contributed to the article and approved the submitted version.

## Funding

This research was supported by Grant 81770963 from National Natural Science Foundation of China (to FP) and Grant 2018YFA0800801 from National Key Research and Development Program of China (to LJ).

## Conflict of interest

The authors declare that the research was conducted in the absence of any commercial or financial relationships that could be construed as a potential conflict of interest.

## Publisher's note

All claims expressed in this article are solely those of the authors and do not necessarily represent those of their affiliated organizations, or those of the publisher, the editors and the reviewers. Any product that may be evaluated in this article, or claim that may be made by its manufacturer, is not guaranteed or endorsed by the publisher.
