# Supplementary material for: Decrease of FZD4 exon 1 methylation in probands from FZD4-associated FEVR family of phenotypic heterogeneity
Source: Front Med (Lausanne). 2022 Oct 24;9:976520. doi: 10.3389/fmed.2022.976520 (PMC9638120; doi:10.3389/fmed.2022.976520)
Supplement: Supplementary file 1 [file Table_1.DOCX]

Supplementary Material


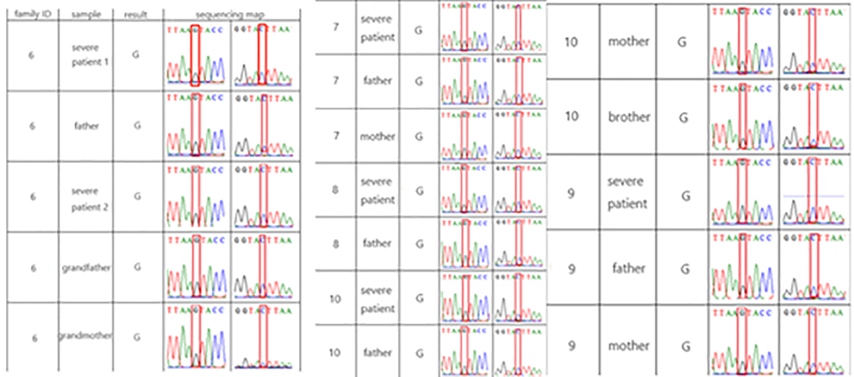


**Supplementary Figure 1.** Sanger sequencing results of family NO. 6, 7, 8, 9, 10 were presented above. All samples detected at the polymorphism site rs10128621 were base G.
